# Network Analysis of Oyster Transcriptome Revealed a Cascade of Cellular Responses during Recovery after Heat Shock

**DOI:** 10.1371/journal.pone.0035484

**Published:** 2012-04-18

**Authors:** Lingling Zhang, Rui Hou, Hailin Su, Xiaoli Hu, Shi Wang, Zhenmin Bao

**Affiliations:** Key Laboratory of Marine Genetics and Breeding, College of Marine Life Sciences, Ocean University of China, Qingdao, China; Auburn University, United States of America

## Abstract

Oysters, as a major group of marine bivalves, can tolerate a wide range of natural and anthropogenic stressors including heat stress. Recent studies have shown that oysters pretreated with heat shock can result in induced heat tolerance. A systematic study of cellular recovery from heat shock may provide insights into the mechanism of acquired thermal tolerance. In this study, we performed the first network analysis of oyster transcriptome by reanalyzing microarray data from a previous study. Network analysis revealed a cascade of cellular responses during oyster recovery after heat shock and identified responsive gene modules and key genes. Our study demonstrates the power of network analysis in a non-model organism with poor gene annotations, which can lead to new discoveries that go beyond the focus on individual genes.

## Introduction

Oysters are a major group of marine bivalves which represent about 8,000 species worldwide [1]. They usually inhabit coastal shallow waters and estuaries, and like other marine ectotherms they can tolerate a wide range of natural and anthropogenic stressors such as thermal fluctuation, anoxia, osmotic change, and a variety of toxicants. Among environmental factors, temperature has long been recognized as a key factor that can potentially influence all physiological processes in marine ectotherms [2]. Oysters can experience rapid and dramatic temperature fluctuations during diurnal/tidal cycles (up to 10∼20°C within a few hours) and seasonal changes (from 0 to 35∼40°C) [3]. It has been shown that thermal tolerance is rather a complex physiological trait which requires the initiation of coordinated cellular responses to thermal stress [4]. Many oyster studies focused on understanding of cellular responses during heat stress [5], [6], whereas the recovery process after heat shock has been less studied. Recent studies have shown that oysters recovered from sublethal heat stress could be more resistant to subsequent thermal stress (i.e. acquired thermal tolerance) [7]. However, most of these studies focused on heat shock proteins (HSPs) or HSP-related proteins [4], [7], [8]. A systematic study of cellular recovery from heat shock would provide new insights into the mechanism of acquired thermal tolerance. Lang et al. [9] have recently performed the transcriptome profiling of selectively bred Pacific oyster (*Crassostrea gigas*) families during recovery after heat shock (RHS) using an oyster cDNA microarray containing 13,752 features [10]. Although they identified ∼110 candidate genes that showed differential expression patterns during RHS, their analytical procedure basically represents a gene-centric approach that focuses on individual genes with high statistical significance. Such approach ignores gene interactions and might suffer from lack of sufficient contextual information for generating scientifically sound hypotheses.

Recent developments in statistical genomics provide a foundation for a shift from the gene-centric to a network- or module-centric approach in microarray data analysis [11]. In addition to determining the roles of individual genes, network analysis enables researchers to study cells as a complex network of biochemical factors. Although network analysis has been widely used in gene expression studies of human and model organisms [12]–[14], little effort has been devoted to expanding its application to the less-studied non-model organisms such as oysters.

Here we present the first network analysis of oyster transcriptome by reanalyzing microarray datasets from Lang et al. [9] to identify gene modules and candidate key genes responsible for oyster RHS.

## Results and Discussion

### Network construction and identification of an RHS-responsive module

In the study of Lang et al. [9], transcriptome profiling was performed using an oyster cDNA microarray for Pacific oyster families that were sampled at different recovery times (1, 3, 6 and 24 hours) after heat shock (40°C for 1 hour). Because barely little difference of gene expression was observed among families in their study, microarray data from these families were combined to increase the power for detection of coexpression patterns in the network analysis.

**Figure 1:**
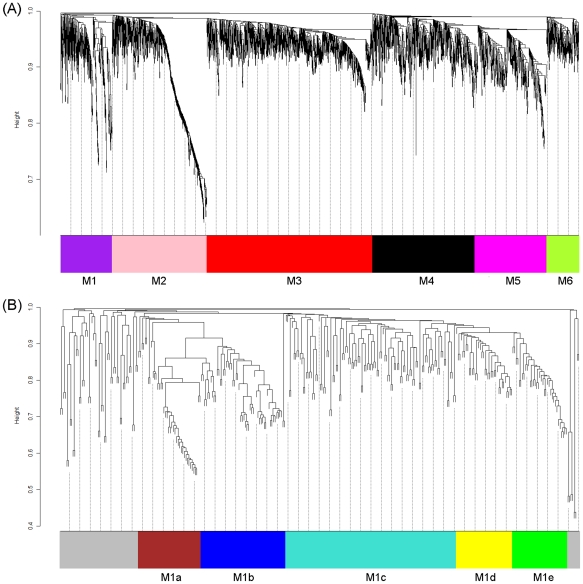
Network analysis of the oyster gill transcriptome during recovery after heat shock (RHS). (A) and subnetwork analysis of the RHS-responsive module M1 (B). Dendrograms are produced by average linkage hierarchical clustering of genes on the basis of topological overlap (see Methods for details). Modules of coexpressed genes are labelled in unique colors. Unassigned genes are labelled in grey.

Network analysis was performed using a recently developed weighted gene co-expression network analysis (WGCNA) method [15] that enables identification of transcriptional modules and key/driver genes within modules based on gene-to-gene correlations across all microarray samples. A total of 60 samples (12 oyster samples×5 time points) were used for calculation of gene-to-gene correlations. The oyster cDNA microarray contained 13,752 probes, of which 3,362 probes representing 1,668 genes passed the quality filter and were included in the network construction. In total, six modules (M1∼M6) containing almost all expressed genes were identified with module size ranging from 211 to 1,075 (Figure 1, Table S1). It is worth mentioning that for genes that had probe replicates, 96% of the replicate probes were assigned to the same module colors, suggesting the high reproducibility of the microarray data as well as the high reliability of network construction. The analysis of variance (ANOVA) revealed that 336 probes were differentially expressed (FDR<0.05) among sampling times (T0, T1, T3, T6 and T24), accounting for ∼10% of all expressed genes. To identify modules responsive to heat stress, enrichment analysis of differentially expressed genes (DEGs) was performed for each module using a hypergeometric test. It turned out that only M1 was significantly enriched with DEGs (*p* = 4e-128). Of the 332 probes in this module, 193 (58%) were DEGs.

### M1 subnetwork

**Figure 2:**
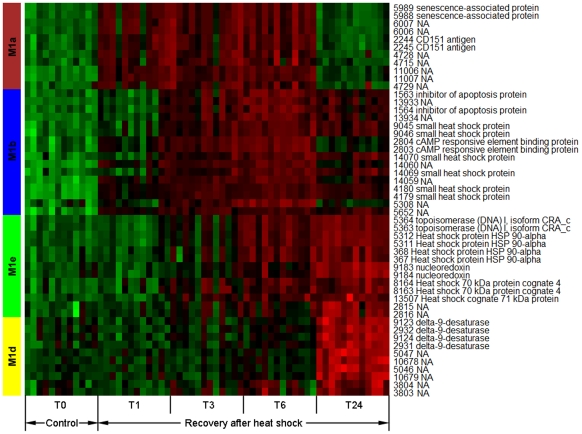
Heat map visualization of coexpression patterns of hub genes in submodules M1a, M1b, M1d and M1e. T0 represents an oyster control without heat treatment, whereas time points T1, T3, T6 and T24 represent recovery time (hour) after heat shock. Probe IDs and their associated gene annotations are shown on the right of the heat map. Red, up-regulation; Green, down-regulation.

In order to gain a better understanding of coexpression patterns in M1, a subnetwork was constructed for M1. As shown in Figure 1, M1 was composed of five submodules (M1a∼M1e). Hub genes (i.e. top 15% genes with high intramodular connectivity) in M1 only distributed in four submodules (M1a, M1b, M1d and M1e). A heat map was constructed for visualization of coexpression patterns of these hub genes in the 4 submodules (Figure 2). Hub genes in M1a showed elevated expression at T1, T3 and T6 after heat shock. Two hub genes in this module were annotated; one was *SAG* (senescence-associated protein) and the other was *CD151* (cluster of differentiation 151). Activation of *SAG* indicates the induction of cellular senescence [16], [17]. It has been shown that senescence and apoptosis can compete with each other in an exclusive way, and senescence can proceed when apoptosis is inhibited [18]. A recent study has revealed that activation of CD151 can facilitate the inhibition of apoptosis possibly through regulation of Bax and Bcl-2 genes [19]. Besides *CD151*, M1a contained other annotated DEGs such as *CDH1* (Cadherin-1), activation of which is also associated with inhibition of apoptosis [20]. Therefore, coexpression patterns observed in M1a may indicate the ongoing transition from apoptosis to cellular senescence. At T24, gene expression in M1a went back to the normal level, possibly suggesting the termination of cellular senescence.

Subsequent to M1a, hub genes in M1b showed responsive expression at T3, T6 and T24 after heat shock. *IAP* (inhibitor of apoptosis protein), *CREB* (cAMP response element-binding protein) and *sHSPs* (small heat shock proteins) are annotated hub genes in this module. IAP blocks apoptosis at the core of the apoptotic machinery by inhibiting effector caspases [21]. Interaction between *IAP* and CREB was demonstrated in a previous study showing that CREB can regulate the promoter activity of *IAP* by binding to the *IAP*'s enhancer sequence [22]. In response to heat stress, sHSPs can stabilize protein conformation, prevent aggregation and thereby maintain the non-native proteins in a competent state for subsequent refolding, which is achieved by other HSPs/chaperones (e.g. HSP70 and HSP90) [23]. In addition, sHSPs also play an important role in inhibition of apoptosis [24]–[26]. It has been proposed that HSP-mediated regulation of the apoptotic pathways probably constitutes a fundamental protective mechanism that decreases cellular sensitivity to damaging events to allow cells to escape the otherwise inevitable engagement of apoptosis [27]. Therefore, M1b is enriched with genes functioning in inhibition of apoptosis as well as stabilization of protein conformation.

M1e contained hub genes with increased expression at T6 and T24, indicating a later response during RHS than M1a and M1b. *HSP70* and *HSP90* are dominant hub genes in this module, which are well known as molecular chaperones that help in the refolding of misfolded proteins and assist in their elimination if they become irreversibly damaged. Elevated expression of *TOP1* (topoisomerase I) was observed in this module, which involves in regulation of DNA supercoiling that might be accumulated during rapid induction of the heat-shock genes [28]. It has been shown that TOP1 plays an important role in the acquisition of thermotolerance probably by preventing inhibition of further transcription of *HSPs* caused by hyper negative supercoiling [29]. Expression of *NRX* (nucleoredoxin) was also increased in this module. NRXs are a novel member of thioredoxin family. Members of the thioredoxin family have been shown to function as facilitators and regulators of protein folding [30]. Taken together, it seems that M1e is enriched with protein-refolding associated genes.

M1d was composed of hub genes with increased expression only at T24. *ACOD* (delta-9-desaturase) is the only annotated hub gene in this module, which functions in conversion of saturated fatty acids to monounsaturated fatty acids. Up-regulation of *ACOD* indicated the increase of unsaturation of lipid membrane (i.e. increased membrane fluidity) during RHS. Cell membranes are known to be a highly sensitive monitor of the most diverse environmental changes. The unsaturation level of membranes is involved in the transduction of thermal stress into cellular signals, thus affecting the general stress-response mechanisms [31]. The increase of membrane fluidity can elevate the sensitivity of cells to heat [32], implying that when oysters are subject to subsequent exposure to heat stress, the cells might respond at a temperature lower than the original threshold, causing the repair system to function faster than previously. This speculation may well explain the previous observations that oysters pretreated with heat shock can result in induced heat tolerance [7]. It has also been shown that desaturase activation or hyper-induction plays an important role in the response to heat stress in certain thermotolerant yeast and bacterial strains [33], [34].

**Figure 3:**
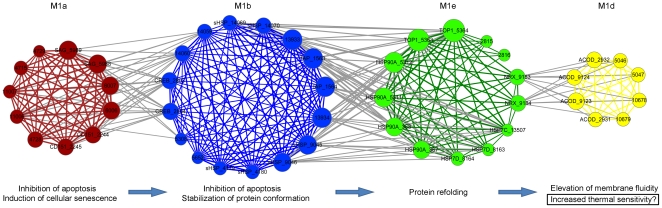
Network visualization of submodule interactions. Each node represents a hub gene. Hub genes derived from the same submodule are labelled in the same color. Hub genes are connected by an edge if the topological overlap between them is more than 0.13. Interactions (i.e. edges) between modules are labelled in grey.

Network visualization of the four submodules revealed a cascade of cellular responses (M1a→M1b→M1e→M1d) during RHS. As shown in Figure 3, inter-modular interactions only occurred between adjacent modules. For example, M1b is the only module that interacts with M1a, but no interactions between M1a and the other two modules. According to Figure 3, the scenario of cellular responses during oyster RHS is likely to be as follows: (i) after heat shock, cellular senescence was induced accompanied by inhibition of apoptosis (M1a); (ii) *sHSPs* were expressed to stabilize protein conformation (M1b) and facilitate further protein refolding by HSP70 and HSP90 (M1e); and (iii) then increase of membrane fluidity was induced, which possibly enhanced the sensitivity of cells to subsequent heat stress.

In addition, network analysis enables identification of key genes responsible for module interactions. One advantage of the coexpression network analysis is that it does not rely on the information of gene annotation for identification of key genes in a module, thus providing the opportunity for identification of novel candidate genes in non-model species with poorly characterized genome. For example, from a network perspective, the unannotated gene (probe IDs 14059 and 14060) in M1b seems to be an important hub gene that is responsible for interactions between M1a and M1b. However, this gene could be possibly overlooked by the traditional analysis methods since no annotation has been assigned to this gene. Network analysis also revealed that *NRX* and *ACOD* are important hub genes that maintain the interactions between M1e and M1d. Although there is no documented direct interaction between these two genes, it might be worthy of further investigation due to the vital role of ACOD in maintaining the fluidity of lipid membrane.

In summary, we performed the first network analysis of oyster transcriptome by reanalyzing microarray datasets from Lang et al. [9]. Network analysis revealed a cascade of cellular responses during oyster RHS and identified responsive gene modules and candidate key genes. Our study demonstrates the power of network analysis in a non-model organism with poor gene annotations, which can lead to new exciting discoveries that go beyond the focus on individual genes.

## Materials and Methods

### Ethics Statement

Not applicable. Our research did not involve human participants or samples.

### Microarray data acquisition

The vsn-transformed microarray data from Lang et al. [9] were downloaded from the Gene Expression Ominibus website (http://www.ncbi.nlm.nih.gov/projects/geo; Series GSE12070, GSM304764∼GSM304823).

### Probe annotation

In order to increase the probe annotation rate, 206,388 ESTs and 1,080,743 raw reads from a 454 sequencing of *Crassostrea gigas* transcriptome project (SRA accession no. SRX032364 and SRX032365) were downloaded from NCBI databases and then assembled using the Newbler v2.3 program (Roche) with default parameter settings. The probes that can be unambiguously mapped to the assembled isotigs were annotated by BlastX the corresponding isotigs against the Nr and SwissProt databases with an e-value threshold of 1E-6. For the remaining probes, they were directly compared against the Nr and SwissProt databases using BlastX with the same e-value threshold. To increase the computational speed, BlastX searches were limited to the first 20 significant hits for each query with non-characterized matching entries (e.g. hypothetical genes/proteins) excluded. Gene names were assigned to each probe based on the best Blast hit, and the corresponding information was provided in Table S1.

### Microarray data pre-processing

The oyster cDNA microarray contains 13,752 probes, of which 3,362 passed the previously defined signal-intensity filter [9] and were included in the subsequent analysis. Outlier values for each gene were removed based on the Grubbs' test (*p*<0.05).

### Gene network construction

Gene network was constructed using the R package WGCNA following the procedure described in [35]. Here we chose a power of eleven so that the resulting networks exhibited approximate scale-free topology (model fitting index R∧2 = 0.71). Next, all genes were hierarchically clustered based on dissimilarity measure of topological overlap which measures inter-connectedness for a pair of genes [15]. The resulting gene dendrogram was used for module detection using the Dynamic Tree Cut method (minimum module size = 80 and cutting height = 0.995) [36]. Fine cutting (minimum module size = 30 and cutting height = 0.997) was further performed for the module of interest.

### Identification of RHS-responsive modules

Differential expression analysis among sampling times was conducted for each probe using analysis of variance (ANOVA). To account for multiple tests, false discovery rate (FDR) was calculated using the qvalue package [37]. Only probes with *q*<0.05 were considered to be differentially expressed. To identify the RHS-responsive module, overrepresentation analysis of DGEs was performed for each module using a hypergeometric test (*p*<0.05).

### Hub gene selection and visualization

Hub genes refer to highly connected genes in a module [15]. They can be determined by calculating the intramodular connectivity *K_in_*, which is a measure of a gene's connection strength to other genes in a module. In this study, top 15% genes with high *K_in_* were considered as hub genes for a given module. Coexpression patterns and interactions of hub genes were visualized using the heat map and Cytoscape [38], respectively.

## Supporting Information

Table S1 **Combined results of probe annotation, WGCNA analysis and differential expression analysis.**
(XLS)
